# On-Farm Welfare Assessment Protocol for Adult Dairy Goats in Intensive Production Systems

**DOI:** 10.3390/ani5040393

**Published:** 2015-09-25

**Authors:** Monica Battini, George Stilwell, Ana Vieira, Sara Barbieri, Elisabetta Canali, Silvana Mattiello

**Affiliations:** 1 Department of Veterinary Science and Public Health (DIVET), Università degli Studi di Milano, Via G. Celoria, Milan 20133, Italy; E-Mails: sara.barbieri@unimi.it (S.B.); elisabetta.canali@unimi.it (E.C.); silvana.mattiello@unimi.it (S.M.); 2 Faculdade Medicina Veterinária, Universidade de Lisboa, Avenida da Universidade Técnica-Pólo Universitário Alto da Ajuda, Lisbon 1300-477, Portugal; E-Mails: stilwell@fmv.ulisboa.pt (G.S.); ana.lopesvieira@gmail.com (A.V.)

**Keywords:** dairy goat, animal welfare, on-farm welfare assessment, animal-based indicator

## Abstract

**Simple Summary:**

The Animal Welfare Indicators (AWIN) project developed a practical welfare assessment protocol for lactating dairy goats in intensive husbandry systems, using animal-based indicators that cover the whole multidimensional concept of animal welfare. The strict collaboration between scientists and stakeholders resulted in an easy-to-use protocol that provides farmers or veterinarians with comprehensive but clear feedback on the welfare status of the herd in less than three hours. The protocol, which highlights key points and motivates farmers to achieve improvements, has received much attention from interested parties.

**Abstract:**

Within the European AWIN project, a protocol for assessing dairy goats’ welfare on the farm was developed. Starting from a literature review, a prototype including animal-based indicators covering four welfare principles and 12 welfare criteria was set up. The prototype was tested in 60 farms for validity, reliability, and feasibility. After testing the prototype, a two-level assessment protocol was proposed in order to increase acceptability among stakeholders. The first level offers a more general overview of the welfare status, based on group assessment of a few indicators (e.g., hair coat condition, latency to the first contact test, severe lameness, Qualitative Behavior Assessment), with no or minimal handling of goats and short assessment time required. The second level starts if welfare problems are encountered in the first level and adds a comprehensive and detailed individual evaluation (e.g., Body Condition Score, udder asymmetry, overgrown claws), supported by an effective sampling strategy. The assessment can be carried out using the AWIN Goat app. The app results in a clear visual output, which provides positive feedback on welfare conditions in comparison with a benchmark of a reference population. The protocol may be a valuable tool for both veterinarians and technicians and a self-assessment instrument for farmers.

## 1. Introduction

The relevance of animal welfare in animal production systems has been increasing over the last decades, due to both economic and ethical reasons. Several examples are reported by [1] supporting the presence of a positive relationship between animal welfare and milk yield, milk composition, and conception rate at first service in cattle. Research in goats has also demonstrated that good welfare conditions lead to higher productivity and profitability [2].

Furthermore, consumers have been focusing their attention on animal welfare as it is acknowledged to be a food quality attribute, like nutritional, safety, and organoleptic properties, but also as an ethical concern [3,4].

In response to this demand, assurance schemes for high-quality animal products, in terms of health, safety, and respect for animal welfare [5], have increased. Therefore, animal welfare assessment at the farm level has become one of the most debated issues in the field of animal husbandry. This issue has been receiving attention in specific working groups, such as the European Action 846 of the COST Framework “Measuring and monitoring farm animal welfare” [6], which in 2004 led to the funding of the EU project Welfare Quality^®^, with the aim of developing on-farm welfare assessment schemes. The output of this project consists of welfare assessment protocols for species with very high economic relevance, *i.e.*, cattle, pigs, and poultry. Because welfare assessment for other farmed species (*i.e.*, sheep, goats, horses, donkeys, and turkeys) was not addressed, the Animal Welfare Indicators (AWIN) project was funded by the EC in 2011. These species are reared with broadly different systems, ranging from very intensive to pasture-based systems, and different production settings, ranging from intensive milk production to extensive meat production or working animals. For goats, the protocol was developed for lactating adult dairy goats kept under intensive or semi-intensive production systems, because they represent the largest animal category in the most widespread goat farming systems in Europe [7]. Furthermore, intensive production of dairy goats is relatively new and little is known about its effect on goats’ health and welfare, apart from a few specific studies [8,9].

This paper describes the approach and the methodology followed to develop the protocol and presents the AWIN welfare assessment protocol for lactating dairy goats in intensive or semi-intensive production systems developed in the frame of the AWIN project by a team of researchers in Portugal and Italy.

## 2. Material and Methods

The following steps were achieved in order to develop the welfare assessment protocol: (1) selection of a set of candidate welfare indicators; (2) stakeholder consultation; (3) prototype testing; and (4) development of a sampling strategy.

### 2.1. Selection of Candidate Welfare Indicators

Welfare indicators should be valid, reliable, and practically feasible in the field [10,11]. Two broad categories of indicators can be used to assess animal welfare at the farm level: (a) animal-based indicators (e.g., behavioral measurements, productivity, and health records); and (b) resource- and management-based influencing factors (e.g., stocking density, feeding regime, milking procedures, *etc.*) [12]. Resource- and management-based indicators have been more frequently included in welfare assessment protocols, because measurements are usually objective, quick, and easy (e.g., Animal Needs Index TGI 35L) [13]. However, the present approach suggested by European Food Safety Authority [14] emphasizes the role of animal-based indicators, as they seem more appropriate for measuring the actual welfare state of the animals, whereas resource- and management-based indicators should be considered as risk factors that might affect welfare. For these reasons, animal-based indicators were preferred for inclusion in the AWIN protocols, although some resource-based indicators were also included when no animal-based indicator was available to assess specific aspects.

As a starting point, a group of experts reviewed the relevant scientific literature to select promising animal-based indicators to be included in the protocol [11]. Indicators were checked for their validity, reliability, and feasibility, identifying gaps in current knowledge, and classified according to the four principles and the 12 criteria developed by Welfare Quality^®^: (1) Good feeding (appropriate nutrition, absence of prolonged thirst); (2) Good housing (comfort around resting, thermal comfort, ease of movement); (3) Good health (absence of injuries, absence of disease, absence of pain and pain induced by management procedures); and (4) Appropriate behavior (expression of social behavior, expression of other behaviors, good human-animal relationship, positive emotional state). As a result of this review, at least one indicator for each welfare criterion was selected to be included in the prototype protocol. Some indicators (e.g., body condition score and hair coat condition) seemed to be promising for providing information on more than one welfare criterion.

**Figure 1:**
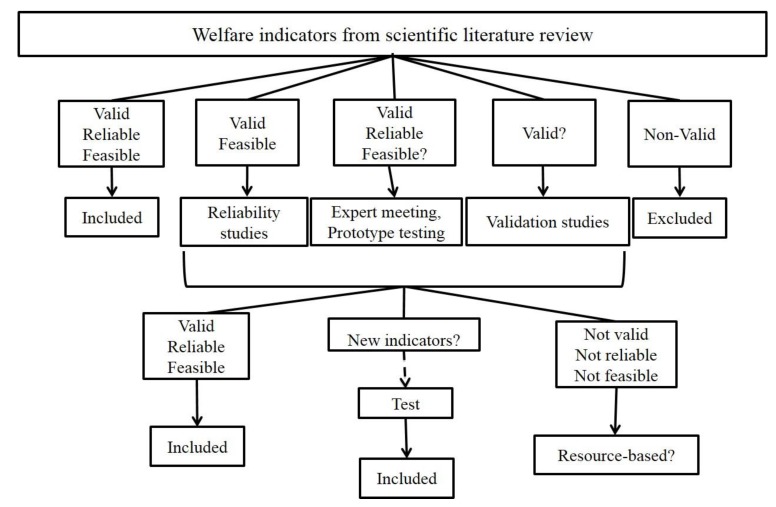
Characteristics and process to identify promising animal-based indicators.

AWIN scientists developed a research action plan to address the lack of knowledge regarding the validity, reliability, and feasibility of single promising indicators where this was not reported in the literature (Figure 1). The validity of new indicators was assessed in specific validation studies, some of which have already been published (hair coat condition: [15]; thermal stress: [16]). In some cases, existing indicators have been modified in order to increase their on-farm feasibility (Body Condition Score: [17]).

### 2.2. Stakeholder Consultation

To develop the welfare assessment protocols, stakeholders’ perception of the selected indicators was taken into consideration. The purpose of involving the stakeholders was to increase the acceptability of the project outcomes through stimulation of a multidisciplinary dialogue, and to identify solutions to potential barriers to the subsequent application of the protocols in practice. Stakeholders’ opinion and farmers’ experience were crucial for the successful implementation of the protocols. Thirty-six people from different European and non-European countries (e.g., Italy, Spain, Portugal, UK, United States, Brazil, Mexico, and Australia) answered an online survey translated into five languages (http://www.questionari.unimi.it/awin/), which was available for 15 months on the AWIN project website and other institutional sites (e.g., Food and Agriculture Organization). The stakeholders were asked to express their feeling about the needs of farmed dairy goats and about the most suitable indicators to identify them. Furthermore, stakeholder opinions were collected during two meetings held at two national goat conferences, where attendees were asked to fill out a questionnaire (21 out of 40 people surveyed in Italy, and 11 out of 21 people surveyed in Portugal responded to the questionnaire). In each meeting, the importance of using animal-based indicators for welfare assessment was initially discussed, and the ongoing studies on validity, reliability, and feasibility were presented. After this, the attendees answered a questionnaire on: (a) the goals of a goat welfare assessment tool; (b) who (role/profession) should perform the assessment; (c) what unintended outcomes should be avoided; and (d) the acceptable length of time for on-farm welfare assessment. Furthermore, the participants ranked, according to their perceived importance, the indicators within each criterion, and provided tentative prevalence of the indicators at farm level, and the highest prevalence they would consider acceptable.

Both the online survey and questionnaire involved veterinarians, farmers, and technicians (online survey: 30.8%, 48.7%, and 20.5%, respectively; questionnaire: 25.0%, 46.9%, and 28.1%, respectively). Part of the results of these surveys has already been published [18].

### 2.3. Testing Prototypes

From the abovementioned process, a list of 25 welfare indicators was generated and included in a prototype protocol. For each of these indicators, specific interactive learning material was prepared and used to provide a common training to the assessors. The learning material consisted of a PowerPoint file starting with a brief description of the indicator, followed by a detailed explanation of the assessing and scoring procedures. Pictures and video-clips were given as examples in order to simulate on-farm conditions and therefore improve the understanding and assessment of the indicator. At the end of the learning process, the level of knowledge was tested to determine if the assessors were ready to perform the farm visits. This preliminary phase was followed by a one-week training period, which included both theoretical and practical sessions, in order to reach an agreement among assessors. The assessment was repeated until all the assessors agreed on the evaluation of each indicator.

In Portugal, farms for prototype testing were randomly selected from a national database of Direcção-Geral de Alimentação e Veterinária, whereas in Italy farms were selected with the support of the S.A.T.A. (Technical Advice Service for Farmers) of the Lombardy region, where the majority of intensive dairy goat farms are concentrated. Because in Portugal small, intensive dairy farms are very rare, only those with more than 50 adult goats were considered. Italian farms are smaller than Portuguese ones, so small farms (<50 adult goats) were also recruited. On the contrary, very large farms are rare in Italy; therefore, this category was tested only in Portugal. In both countries, farms mainly housed cosmopolitan breeds (Saanen and Alpine) and their crossbreeds. Some local breeds were also present (e.g., Nubian, Murciana, Serrana, Frisa Valtellinese). Most farms kept goats permanently indoors in straw litter. Goats were fed by Total Mixed Ration and water was always available. In all farms kids were removed after birth. The prototype was tested in 60 intensive dairy goat farms (30 in Italy and 30 in Portugal) with different sizes (mean ± SE 192.25 ± 29.22; min 14; max 1147 lactating goats) and characteristics (e.g., presence/absence of feeding rack, stocking density, feeding space, *etc.*), in order to test the feasibility of the selected indicators in different farming conditions. In order to check inter-observer reliability, 20 farms out of the 60 in assessment were selected by convenience to be visited by two trained assessors at the same time. Inter-observer reliability was excellent for all indicators included in the protocol, either collected at group level (Spearman’s correlations; *p* < 0.001) or at individual level (index of concordance ranging from 80.27% to 100%) [19].

Additionally, in order to check consistency across seasons, 20 farms out of the total number were visited two times (in winter and in summer) by the same observer. Investigations on the correlation of animal-based welfare indicators between two consecutive farm visits highlighted an overall consistency of all the indicators included in the final protocol [20].

In order to facilitate collection and analysis of data collected during on-farm inspections, a digitalized data collection system was used, starting from Open Data Kit (ODK), a free and open-source set of tools that manages mobile data collection solutions, developed by the University of Washington’s Department of Computer Science and Engineering [21]. This system also allowed us to automatically record the time needed to assess each indicator, providing important information about the feasibility of the prototype [22].

### 2.4. Sampling Strategy

For the development of a practical welfare assessment scheme, it is necessary to develop a sampling strategy that makes the protocol feasible in terms of time and costs. For this reason, preliminary observations were carried out on the whole herd in four goat farms (total of 657 adult lactating goats), in order to determine the optimal sampling strategy on the number of pens to be assessed [23]. Furthermore, in order to validate the previous results, another study based on observations conducted on the whole herd in 12 intensive dairy goat farms (total of 4228 adult lactating goats) was conducted. In this study five different sampling strategies (every strategy considered a different number of pens to be chosen for assessment) were compared by means of their minimum mean-square error. On the basis of the results, a sampling strategy for pen assessment was defined [24]. As some indicators have to be collected on individual animals, in the study in which 12 farms were assessed, the minimum sample of individual animals to be evaluated was also calculated. Assuming a 50% prevalence of the indicators in the population (the worst possible condition in terms of animals needed for sampling purposes), and a 10% accuracy (expressed as a percentage relative index, indicating how close a sample-based parameter estimator is to the true data population value [25]), two sampling schemes for individual animals were developed: one based on a 95% confidence interval (IC) (suggested sample), and another based on a 90% IC (minimum sample). Taking into account the fact that previous research had shown that some of the selected indicators were correlated with the order of entry to the milking parlor [26,27], systematic selection of animals is recommended, as this presents a lower minimum mean-square error.

## 3. Results

The final protocol derives from the results of the literature review [18], the research studies carried out by the AWIN project, the feedback from the stakeholders, the prevalence recorded for each indicator during prototype testing, and simulations for the sampling strategy.

After prototype testing, seven indicators were excluded from the final protocol due to very low prevalence (abnormal lying posture, vulvar discharge), scarce reliability (lesions, kneeling in the pen), low validity (cleanliness), or low feasibility (avoidance distance test, knee calluses) [19,20,21,22]. Cleanliness was replaced by a resource-based indicator (bedding), in order to cover the “comfort around resting” criterion. Thermal stress indicators (panting score and shivering score) [16] were merged into one indicator.

A two-level approach was chosen to increase feasibility and acceptability among stakeholders without losing scientific validity. The protocol offers, at the first level, a quick screening, consisting of a selection of robust, feasible, and, in some cases, multi-criteria animal-based indicators, which can be readily applied and require no handling of the animals. Depending on the outcome of the first level welfare assessment, a second level, consisting of a more comprehensive and in-depth assessment, may be recommended. At the second level, animals are often handled, but the welfare assessment is still feasible.

**Table 1:** General description and characteristics of the welfare indicators of the AWIN welfare assessment protocol for goats. A more detailed description is available in the protocol (AWIN, 2015).

Indicator	Principle ^a^	Criteria	Description	Measurement/Scoring	Level	Where to Assess ^b^	Target ^c^
Abscesses *	Health	Absence of disease	The presence of external abscesses is recorded.	Presence/absence	1st & 2nd	O/R	G/I
Bedding	Housing	Comfort around resting	The quantity and quality of the bedding in the pen is evaluated.	Quantity: sufficient/insufficient; quality: clean/dirty	1st & 2nd	I	RB
Body Condition Score	Feeding/Health	Appropriate nutrition/Absence of disease	Body Condition Score is visually assessed on individual goats, using a three-level visual scoring method.	Very thin/normal/very fat	2nd	R	I
Fecal soiling	Health	Absence of disease	The presence of manure below the tail head is visually assessed on individual goats, as a sign of diarrhea.	Presence/absence	2nd	R	I
Hair coat condition	Feeding/Health	Appropriate nutrition/Absence of disease	The number of goats with poor hair coat condition (described as: matted, rough, scurfy, uneven, shaggy hair coat, frequently longer than normal) is recorded.	Number of goats	1st & 2nd	O	G
Improper disbudding	Health	Absence of pain and pain induced by management procedures	The number of goats showing presence of residual horns (scurs) is recorded.	Number of goats	1st & 2nd	O	G
Kneeling at the feeding rack	Housing	Ease of movement	The number of kneeling goats (front legs flexed, the rear up compared to other goats) is counted while they are at the feeding rack.	Number of goats	1st & 2nd	O	G
Latency to the first contact test	Behavior	Good human-animal relationship	The latency from the time the assessor stops in a pre-determined starting place in the pen and the contact with the first goat that nuzzles or touches any part of the assessor’s body is recorded (max time: 300 s).	Time elapsed	1st & 2nd	I	G
Nasal discharge	Health	Absence of disease	The presence of any mucous or purulent discharge from the nose is visually assessed on individual goats.	Presence/absence	2nd	R	I
Oblivion	Health	Absence of disease/Expression of other behaviors	The number of oblivious goats is recorded. An oblivious goat is defined as an animal that is physically or mentally isolated from the group.	Number of goats	1st & 2nd	O	G
Ocular discharge	Health	Absence of disease	The presence of clearly visible flow from one or two eyes is visually assessed on individual goats.	Presence/absence	2nd	R	I
Overgrown claws	Health	Absence of injuries	The presence of rear claws that are deformed and/or with excess horn tissue is visually assessed on individual goats.	Acceptable/not acceptable	2nd	R	I
Qualitative Behavior Assessment	Behavior	Positive emotional state	The assessor integrates perceived details of behavior, posture, and context into the summarization of an animal’s style of behaving, or “body language”, using descriptors such as “aggressive”, “fearful”, “frustrated,” or “content.”	Scores on visual analogue scale	1st & 2nd	O	G
Queuing at drinking	Feeding/Behavior	Absence of prolonged thirst/Expression of social behavior	The number of goats queuing at the drinker is counted during feeding time, using a scan sampling method.	Number of goats	1st & 2nd	O	G
Queuing at feeding	Feeding/Behavior	Appropriate nutrition/Expression of social behavior	The number of goats queuing at the feed rack is counted during feeding time, using a scan sampling method.	Number of goats	1st & 2nd	O	G
Severe lameness	Health	Absence of injuries/Absence of pain and pain induced by management procedures	Goats are moved in the pen and the number of severely lame animals (based on abnormal gait, head nodding, spine curvature, and presence of kneeling in places other than the feeding rack) is counted.	Number of goats	1st & 2nd	I	G
Thermal stress	Housing	Thermal comfort	The number of goats showing heat (accelerated respiration rate with open mouth and excessive salivation) or cold (hair horripilation or shivering) stress signs is counted.	Number of goats	1st & 2nd	O	G
Udder asymmetry	Health	Absence of injuries	The presence of asymmetric udders (in which one half is at least 25% longer than the other, excluding the teats) is visually assessed on individual goats.	Presence/absence	2nd	R	I

**^a^** Feeding = Good feeding; Housing = Good housing; Health = Good health; Behavior = Appropriate behavior; **^b^** O = Outside the pen; I = Inside the pen; R = Restrained at feed rack or milking parlor; **^c^** G = Group; I = Individual; RB = Resource-based; * Abscesses are evaluated both in the 1st level (only front part of the animals, observed from outside the pen, in the whole group) and in the 2nd level (on individually restrained animals, both front and rear regions).

### 3.1. First-Level Welfare Assessment Protocol

The protocol consists of 12 welfare indicators, which cover all the principles and criteria developed by Welfare Quality^®^ (Table 1). All indicators are animal-based, except for bedding. Detailed information on description, assessment, and method of scoring of each indicator can be found in the AWIN welfare assessment protocol for goats [24].

**Figure 2:**
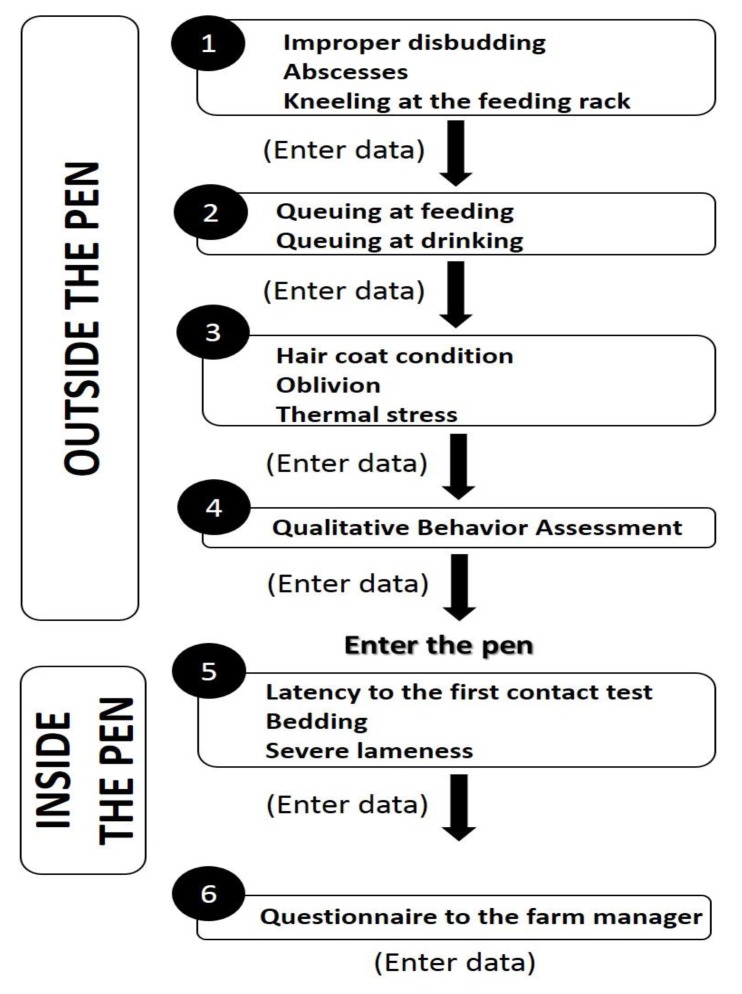
Flow of the first-level welfare assessment.

In the first level, indicators are recorded at group level by a single assessor and no individual restraint of the animals is required. The assessment starts at the time of feed distribution (main meal) from outside the pen and continues inside the pen. The different indicators should be collected following a pre-established fixed order (Figure 2), to avoid biased results on behavioral indicators.

Only one pen has to be assessed in the first-level welfare assessment protocol, even in farms with multiple pens. The time needed for this assessment is approximately 90 min. In order to increase the sensitivity of the assessment [1], the pen considered as presenting the potentially greatest risk for welfare has to be selected by the assessor. This selection takes into consideration the following aspects: highest density, lower feeding space/animal ratio, lower drinking place/animal ratio, and presence of both horned and hornless goats in the same pen. If all pens are in similar conditions, one random pen should be selected. No buck should be present in the pen at the time of the assessment, as their presence may affect the behavior of the does. Infirmary, culling, quarantine, or maternity pens should never be assessed.

At the end of the first level data collection, a clear and immediate output for the farmers is generated (see Section 3.4) by a specific app (AWIN Goats, freely available on Google Play Store and App Store) for tablet or smartphone. The app was developed in order to improve the digitalized data collection system used for prototype testing [21].

After applying the protocol, the farm manager is asked to answer a questionnaire. The aim of the questionnaire is to gather general information about the farm characteristics, housing structures, management procedure, and main productive and reproductive data (e.g., milk production, somatic cell count, average age of lactating goats, and number of deliveries per goat). It is mandatory that the farm manager is interviewed at the end of data collection, to avoid the assessor being influenced by the farm manager’s attitude during the collection of the indicators. The answers to the questionnaire are not included in the outcome, but they can be used for identifying risk factors and understanding the underlying reasons for any welfare problem that may arise from the protocol application [28].

### 3.2. From First- to Second-Level Welfare Assessment Protocol

Several conditions were set to establish whether a farm needs a deeper welfare assessment.

First of all, performance of the second-level assessment is recommended whenever there is noncompliance with the local legislation on animal protection and welfare.

The second level is also recommended when the first-level assessment suggests the presence of some possible welfare issues (nutritional or health problems, behavioral problems, and fear of humans). Specifically, the second-level welfare assessment is suggested when the result of the assessment for some previously selected welfare indicators (abscesses, improper disbudding, hair coat condition, severe lameness, queuing at feeding, queuing at drinking) shows that the within-farm proportion of animals is lower than the proportion of animals observed in the worst 5% of the reference population, or when no goats touch the assessor during the 300 s of the “latency to the first contact test.”

### 3.3. Second-Level Welfare Assessment Protocol

In this second level, 18 indicators have to be evaluated. Eleven of them are collected following the same methodology described in the first-level protocol, whereas seven have to be evaluated on individual animals.

In this level, if more than one pen is present, more pens should be evaluated. If there are between two and seven pens, at least two pens should be assessed. If there are more than seven pens, at least three pens should be assessed and, if more than 11 pens are present (excluding infirmary, culling, quarantine, or maternity pens), at least 25% of the pens have to be assessed and the assessment will require two or more days. As the presence of two assessors is always required for the second step, during group assessment the two assessors will evaluate one pen each.

For the evaluation of indicators on individual animals, goats need to be restrained and handled, although disturbance to the animals is kept to a minimum. The location for individual examination of goats may vary, depending on the farm characteristics: if goats can be restrained at the feeding rack in their home pen, this is preferable, although the assessment should not be performed during feeding time. If this is not possible, animals can be inspected in the milking parlor. The choice of where to assess the animals should be previously agreed with the farmer. The individual assessment is carried out by two assessors at the same time: one assessor will evaluate the front (for ocular discharges, nasal discharges, and abscesses on the head, neck, and shoulders) and the other the rear part (for Body Condition Score, overgrown claws, fecal soiling, udder asymmetry, and abscesses on the hindquarters) of the goats.

**Figure 3:**
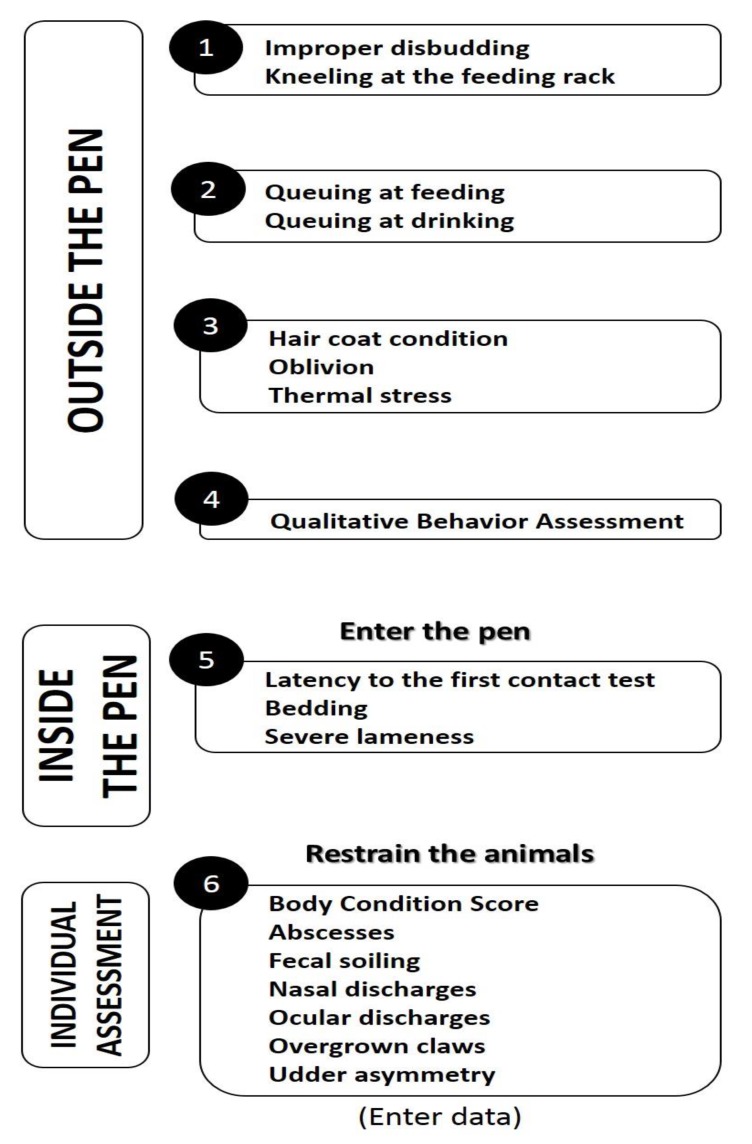
Flow of the second-level welfare assessment.

As in the first level, the different indicators should be collected following a pre-established fixed order (Figure 3).

The time needed for assessing one or two pens at the same time is approximately 90 min, as in the first level. For individual assessment, approximately 30–45 s per goat are required. Assuming a 50% prevalence of the indicators in the population (the worst possible condition), the number of goats to be assessed is determined on the basis of the number of lactating goats on the farm, in order to have 10% accuracy and a 95% (suggested sample) or 90% IC (minimum sample) (Table 2). If, in a farm, for example, there is only one pen with 100 lactating goats, then it is recommended to sample 49 goats. This means that, when the animals are in the milking parlor or at the feeding rack, about one out of every two goats should be selected for the assessment.

**Table 2:** Suggested and minimum sample size of individual goats to be assessed in the second-level AWIN welfare assessment protocol.

Farm Size—Number of Lactating Goats	Suggested Sample ^1^	Minimum Sample ^2^
<15	all animals	all animals
15–19	13	13
20–24	17	16
25–29	20	19
30–34	23	21
35–39	26	24
40–44	29	26
45–49	31	28
50–59	33	29
60–69	37	32
70–79	41	35
80–89	44	37
90–99	47	39
100–124	49	41
125–149	55	44
150–174	59	47
175–199	63	49
200–224	65	51
225–249	68	53
250–299	70	54
300–349	73	56
350–399	76	57
400–449	78	57
450–499	80	58
500–599	81	59
600–699	83	60
700–799	85	61
800–899	86	62
900–999	87	63
1000–1099	88	63
1100–1299	89	64
1300–1499	90	65
1500–1699	91	65
1700–1799	91	66
>1800	92	66

**^1^** Assuming a 50% prevalence, IC 95% and accuracy 10%; **^2^** Assuming a 50% prevalence, IC 90%, and accuracy 10%.

### 3.4. Outcome

**Figure 4:**
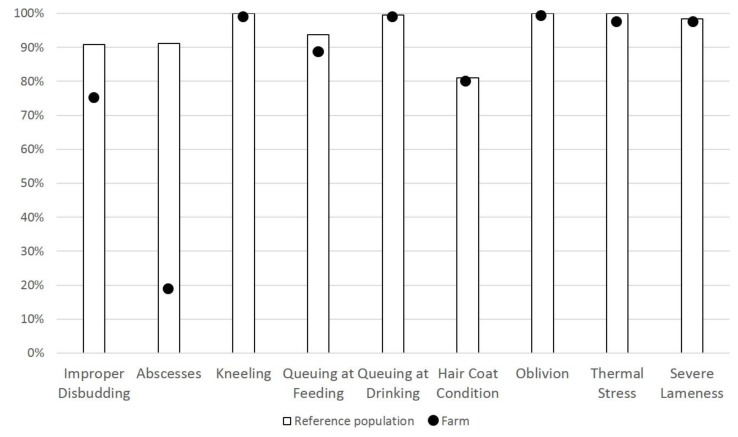
Example of visual output displayed for group indicators in the first-level welfare assessment, excluding Qualitative Behavior Assessment.

The protocol describes how to report data and how to visually represent the results, highlighting positive conditions [24]. For the first-level welfare assessment protocol, the outcome can be automatically generated by the AWINGoat app, and gives an immediate feedback on the welfare of the animals on the farm. All the indicators (except for Qualitative Behavior Assessment and bedding) are displayed in the output and the position of the assessed farm is highlighted in comparison to the median value of the reference population (Figure 4). The data used to calculate the proportion of each indicator are weighted according to the number of goats on the farm.

Qualitative Behavior Assessment is displayed as a separate output (Principal Component Analysis plot generated from the data of the reference population and the assessed farm values) and could be considered a valuable tool when discussing the general demeanor of the animals with the farmer. Currently, the reference population displayed in the outputs refers to data collected during the AWIN project.

## 4. Discussion

The AWIN welfare assessment protocol for goats responds to the initially stated requirements of validity, reliability, and feasibility. All the indicators included in the final protocol were selected based on studies in which their validity and reliability proprieties were established, as was discussed throughout this manuscript. The final protocol includes indicators that cover all the welfare principles and criteria. The indicators excluded were removed because they did not meet the validity, reliability, and feasibility standards needed for their inclusion in a robust welfare assessment scheme. However, no important welfare implication is expected due to their exclusion, because other valid indicators covering the same principles and criteria were retained and, when necessary, they were improved in order to cover all welfare aspects.

In particular, the on-farm feasibility of this protocol is a distinctive feature compared to previous welfare assessment projects [29] and it has being receiving a lot of positive comments and interest from the stakeholders. This is probably due to the fact that there is a good agreement between the choice of the AWIN welfare indicators and the indicators suggested by the stakeholders [18], and also because the time required to perform the assessment is relatively short. In fact, the majority of the stakeholders indicated as acceptable a duration of between 1 and 2 h for group assessment, and 1 to 5 min for individual assessment [18]; therefore, the AWIN protocol falls into an acceptable range of time. Furthermore, it is not invasive and requires no or minimal handling of the animals, especially for the first-level assessment.

Another stakeholders’ request was that the protocol should not be used as a means of controlling the farmers, but rather as an advisory tool, useful to give assistance on how to improve animal management and welfare [18]. This request corresponds very well with the approach used by AWIN, as the outcome is presented in order to give positive feedback to the farmers and encourage further improvements. The AWIN protocol is valuable for providing advice, as it is intended to highlight the strengths and weaknesses of the farm. To this end, the outcome generated by the AWIN Goat app is available immediately after the assessment and it is clear and easy to understand. The comparison provided by AWIN with a reference population is useful for the farmer, in order to have an idea of what can be achieved by other farms, and this is supposed to be a further positive stimulus to improve on-farm management and welfare.

This approach is different from that used in the previous Welfare Quality^®^ project, which was set up mainly for classification of farms into four welfare categories (from not classified to excellent) [30]. The Welfare Quality^®^ output is a numerical value for each welfare criteria, which summarizes the scores deriving from many indicators [31] and assigns the farm to a specific welfare category. This allows the farmer to know whether the welfare on the farm is good or poor, but it does not help with understanding specific welfare problems.

A present limitation of the AWIN protocol is the fact that the reference population is currently based only on 60 farms in two countries. The application of the protocol to a larger number of farms in different countries will allow for updating the reference population in order to have a more reliable benchmark for comparison. In line with the AWIN approach, the reference population should constantly evolve and, consequently, statistical data should be updated, including welfare assessments performed on as many farms as possible using the same common protocol, with the aim of giving an actual picture of the welfare status of goats in intensive farms across Europe.

## 5. Conclusions

The AWIN welfare assessment protocol for dairy goats is designed to enable comparisons among similar production and management systems and is intended to assess goat welfare in order to guide its improvement throughout Europe and elsewhere in the world. It is meant to be comprehensive, but not complex.

The AWIN welfare assessment protocol can also be a valuable tool for farmers who wish to improve the profitability of their dairy farms, pinpointing or preventing issues related to health and welfare that may compromise the status of the herd and its productivity.

The positive feedback that it this protocol is receiving from stakeholders suggests that high acceptability can be expected. This may lead to the adaptation and application of the protocol in different countries and farming conditions. The results will allow for further refining and updating of the reference population in a relatively short period of time.

## References

[1] De Vries M., Bokkers E.A.M., van Schaik G., Engel B., Dijkstra T., de Boer I.J.M. (2014). Exploring the value of routinely collected herd data for estimating dairy cattle welfare. J. Dairy Sci..

[2] Alcedo M.J., Ito K., Maeda K. (2015). Stockmanship competence and its relation to productivity and economic profitability: The context of backyard goat production in the Philippines. Asian-Australas. J. Anim. Sci..

[3] Bredhal M.E., Northern J.R., Boeker A., Normile M.A., Regmi A. (2001). Consumer demand sparks the growth of Quality Assurance Schemes in the European food sector. Changing Structure of Global Food Consumption and Trade.

[4] Broom D.M. (2010). Animal welfare: An aspect of care, sustainability, and food quality required by the public. J Vet. Med. Educ..

[5] Blokhuis H.J., Miele M., Veissier I., Jones R.B. (2013). Improving Farm Animal Welfare: Science and Society Working Together: The Welfare Quality Approach.

[6] Blokhuis H.J., Jones R.B., Geers R., Miele M., Veissier I. (2003). Measuring and monitoring animal welfare: Transparency in the food product quality chain. Anim. Welf..

[7] FAOSTAT 2015. http://faostat3.fao.org/browse/Q/QA/E.

[8] Anzuino K., Bell N.J., Bazeley K.J., Nicol C.J. (2010). Assessment of welfare on 24 commercial UK dairy goat farms based on direct observations. Vet. Rec..

[9] Muri K., Stubsjøen S.M., Valle P.S. (2013). Development and testing of an on-farm welfare assessment protocol for dairy goats. Anim. Welf..

[10] Scott E.M., Nolan A.M., Fitzpatrick J.L. (2001). Conceptual and methodological issues related to welfare assessment: A framework for measurement. Acta. Agr. Scand. Sect. A-Anim. Sci..

[11] Battini M., Vieira A., Barbieri S., Ajuda I., Stilwell G., Mattiello S. (2014). Animal-based indicators for on-farm welfare assessment for dairy goats: A review. J. Dairy Sci..

[12] Main D.C.J., Whay H.R., Green L.E., Webster A.J.F. (2003). Effect of the RSPCA Freedom Food scheme on the welfare of dairy cattle. Vet. Rec..

[13] Bartussek H. (1999). A review of the animal needs index (ANI) for the assessment of animals’ well-being in the housing systems for Austrian proprietary products and legislation. Livest. Prod. Sci..

[14] European Food Safety Authority (2012). Statement on the use of animal-based measures to assess the welfare of animals. EFSA J..

[15] Battini M., Peric T., Ajuda I., Vieira A., Grosso L., Barbieri S., Stilwell G., Prandi A., Comin A., Tubaro F. (2015). Hair coat condition: A valid and reliable indicator for on-farm welfare assessment in adult dairy goats. Small Rumin. Res..

[16] Battini M., Barbieri S., Fioni L., Mattiello S. (2015). Feasibility and validity of animal-based indicators for on-farm welfare assessment of thermal stress in dairy goats. Int. J. Biometeorol..

[17] Vieira A., Brandão S., Monteiro A., Ajuda I., Stilwell G. (2015). Development and validation of a visual body condition scoring system for dairy goats with picture-based training. J. Dairy Sci..

[18] Battini M., Barbieri S., Ferrari L., Bruni G., Zanatta G., Canali E., Mattiello S. (2014). Un protocollo per la valutazione del benessere nella capra: risultati della consultazione con le Parti Sociali in Italia. Large Animal Review.

[19] Conti M. (2014). Ripetibilità Tra Osservatori Di Indicatori Per La Valutazione in Campo Del Benessere Della Capra Da Latte. Master Thesis.

[20] Can E.M.A.V. (2015). Welfare Assessment in Portuguese Dairy Goat Farms: On-Farm Overall Feasibility of an International Prototype. Master Thesis.

[21] Dai F., Dalla Costa E., Battini M., Barbieri S., Minero M., Mattiello S., Canali E. An innovative tool for on-farm data collection and information sharing. Proceedings of the 6th WAFL Conference.

[22] Battini M., Barbieri S., Bruni G., Zanatta G., Mattiello S. Testing the feasibility of a prototype welfare assessment protocol in intensive dairy goat farms. Proceedings of ASPA 21st Congress.

[23] Vieira A., Battini M., Ajuda I., Mattiello S., Stilwell G. Set up of a sampling strategy for the collection of animal-based welfare indicators during milking. Proceedings of the XI International Conference on Goats.

[24] AWIN (2015). AWIN Welfare Assessment Protocol for Goats.

[25] Bartley D.L. (2001). Definition and assessment of sampling and analytical accuracy. Ann. Occup. Hyg..

[26] Main D.C.J., Barker Z.E., Leach K.A., Bell N.J., Whay H.R., Browne W.J. (2010). Sampling strategies for monitoring lameness in dairy cattle. J. Dairy Sci..

[27] Vieira A., Battini M., Ajuda I., Mattiello S., Stilwell G. Is the collection of animal-based welfare indicators during milking affected by the order of goats entry into the milking parlor?. Proceedings of the UFAW International Animal Welfare Science Symposium.

[28] European Food Safety Authority 2009 (2009). Scientific opinion of the panel on animal health and welfare on a request from the commission on the risk assessment of the impact of housing, nutrition and feeding, management and genetic selection on behaviour, fear and pain problems in dairy cows. EFSA J..

[29] Heath C.A.E., Browne W.J., Mullan S., Main D.C.J. (2014). Navigating the iceberg: reducing the number of parameters within the Welfare Quality^®^ assessment protocol for dairy cows. Animal.

[30] Welfare Quality^®^ (2009). Assessment Protocol for Cattle.

[31] Botreau R., Bracke M.B.M., Perny P., Butterworth A., Capdeville J., van Reenen C.G., Veissier I. (2007). Aggregation of measures to produce an overall assessment of animal welfare. Part 2: Analysis of constraints. Animal.

